# Integrated roles of BclA and DD-carboxypeptidase 1 in *Bradyrhizobium* differentiation within NCR-producing and NCR-lacking root nodules

**DOI:** 10.1038/s41598-017-08830-0

**Published:** 2017-08-22

**Authors:** Quentin Barrière, Ibtissem Guefrachi, Djamel Gully, Florian Lamouche, Olivier Pierre, Joël Fardoux, Clémence Chaintreuil, Benoît Alunni, Tatiana Timchenko, Eric Giraud, Peter Mergaert

**Affiliations:** 1 Institute for Integrative Biology of the Cell, UMR9198, CNRS, Université Paris-Sud, CEA, Gif-sur-Yvette, France; 2 Research Unit Biodiversity & Valorization of Arid Areas Bioressources (BVBAA), Faculty of Sciences, Gabès University, Erriadh-Zrig, 6072 Gabès, Tunisia; 3 Laboratoire des Symbioses Tropicales et Méditerranéennes, Institut pour la Recherche et le Développement, UMR IRD/SupAgro/INRA/UM2/CIRAD, Campus International de Baillarguet, TA A-82/J, 34398 Montpellier Cedex 5, France; 4 Present Address: Université de Pau et des Pays de l’Adour, Pau, France; 5 Present Address: Institut Sophia AgroBiotech, Sophia-Antipolis, France

## Abstract

Legumes harbor in their symbiotic nodule organs nitrogen fixing rhizobium bacteria called bacteroids. Some legumes produce Nodule-specific Cysteine-Rich (NCR) peptides in the nodule cells to control the intracellular bacterial population. NCR peptides have antimicrobial activity and drive bacteroids toward terminal differentiation. Other legumes do not produce NCR peptides and their bacteroids are not differentiated. Bradyrhizobia, infecting NCR-producing *Aeschynomene* plants, require the peptide uptake transporter BclA to cope with the NCR peptides as well as a specific peptidoglycan-modifying DD-carboxypeptidase, DD-CPase1. We show that *Bradyrhizobium diazoefficiens* strain USDA110 forms undifferentiated bacteroids in NCR-lacking soybean nodules. Unexpectedly, in *Aeschynomene afraspera* nodules the nitrogen fixing USDA110 bacteroids are hardly differentiated despite the fact that this host produces NCR peptides, suggesting that USDA110 is insensitive to the host peptide effectors and that nitrogen fixation can be uncoupled from differentiation. In agreement with the absence of bacteroid differentiation, USDA110 does not require its *bclA* gene for nitrogen fixing symbiosis with these two host plants. Furthermore, we show that the BclA and DD-CPase1 act independently in the NCR-induced morphological differentiation of bacteroids. Our results suggest that BclA is required to protect the rhizobia against the NCR stress but not to induce the terminal differentiation pathway.

## Introduction

Nodules on the roots of legume plants - and in certain cases also on the stems - are symbiotic organs that house within their cells thousands of nitrogen-fixing rhizobium bacteria, called bacteroids^1, 2^. This intimate co-habitation of eukaryotic cells with bacteria requires adaptations of both the host and the micro-symbiont to maintain a cooperative state to avoid one partner succumbing to the aggression or immune responses of the other. The production of effector peptides, called Nodule-specific Cysteine-Rich (NCR) peptides by the Inverted Repeat Lacking Clade (IRLC) and Dalbergoid legumes is such a host adaptation that helps control the endosymbiotic bacterial population^3–5^. These peptides, related to innate immunity antimicrobial peptides, target the bacteria and induce them into a terminally differentiated state which is characterized by a pronounced cell enlargement resulting in either elongated or spherical bacteroids, depending on the host species. This bacterial cell enlargement is accompanied by a strong polyploidization of the bacterial genome.

Some NCR peptides, mostly cationic ones, have the capacity to kill rhizobia, as well as other bacteria, in agreement with their similarity to antimicrobial peptides such as defensins. Similarly to defensins, the NCR peptides affect bacterial envelope permeability which may lead to bacterial death^3^. In addition to their effects on the bacterial membranes, NCR peptides also have diverse intracellular targets^6^, including the cell cycle machinery, which leads to the bacteroid genome amplification^7^.

NCR-induced bacterial elongation and polyploidization and bacterial survival in the face of NCR toxicity require a SbmA_BacA transmembrane domain-containing peptide importer protein which is able to transport the NCR peptides within the bacterial cytosol. It was proposed that NCR peptide uptake reduces their toxicity by preventing their deleterious effects on the bacterial membrane^8, 9^. Depending on the rhizobium species, two types of NCR transporters have been identified. In *Sinorhizobium meliloti*, the symbiont of the IRLC legumes *Medicago sativa* and *Medicago truncatula*, the NCR transporter, called BacA, consists solely of the SbmA_BacA transmembrane domain and is highly homologous to *E. coli* SbmA which drives peptide transport across the inner membrane by the membrane electrochemical gradient^10^. On the other hand, *Bradyrhizobium* spp. nodulating *Aeschynomene* spp. (Dalbergoids) use for NCR uptake the SbmA_BacA transmembrane domain-containing ABC transporter BclA which has, unlike BacA, a C-terminal ATPase domain and drives transport by ATP hydrolysis^9^. The SbmA_BacA transmembrane domain transporters are wide-spread in bacteria and are not symbiosis-specific proteins. Their bacterial household function remains poorly defined but in *E. coli* or *S. meliloti* they can import a diversity of peptides or peptide derivatives, including antimicrobial peptides that have intracellular targets^11–22^.

Many legumes do not produce NCR peptides and the bacteroids in those legumes do not enlarge, do not become polyploid and are not terminally differentiated^23, 24^. This is the case for legumes of the Robinioids and Millettioid clades such as *Lotus japonicus*, *Phaseolus vulgaris*, *Lablab purpureus*, *Tephrosia vogelii*, *Vigna unguiculata* and soybean (*Glycine max*). Consequently, the *bacA* gene of their symbionts, *Mesorhizobium loti*, *Rhizobium leguminosarum* bv. *phaseoli*, *Rhizobium etli* and *Sinorhizobium fredii*, respectively, can be inactivated without consequences for the symbiosis^1, 8, 25–28^. Based on these case studies, it was proposed that the *bacA* gene in rhizobia has only a symbiotic role when the host plant produces NCR peptides^8^. However, the role of the bradyrhizobial *bclA* gene in interactions with host plants that do not produce NCR peptides has not been studied.

Besides the SbmA_BacA transporters BacA or BclA, a peptidoglycan-modifying enzyme, a specific DD-carboxypeptidase (DD-CPase) enzyme with a peptidoglycan-binding SPOR domain and encoded by the *DD-CPase1* gene, was recently identified in bradyrhizobia as a second rhizobial factor involved in terminal bacteroid differentiation in legumes producing NCR peptides^29^. DD-CPases are periplasmic enzymes that regulate the degree of reticulation in the peptidoglycan layer and thereby affect the physical strength of the bacterial wall. In the NCR-producing legumes *Aeschynomene indica* and *Aeschynomene afraspera*, the bacteroids of the *DD-CPase1* mutant are malformed and hypertrophied. However, in the NCR-lacking soybean, the mutation does not affect the bacteroids^29^.

In this study, we investigated whether *Bradyrhizobium diazoefficiens* (previously *Bradyrhizobium japonicum*) strain USDA110 requires its *bclA* gene in symbiosis with NCR-lacking and NCR-producing legume hosts. We also analyzed how the *bclA* and *DD-CPase1* determinants of terminal bacteroid differentiation interfere with each other, by creating and analyzing double mutants in the *bclA* and *DD-CPase1* genes in *B. diazoefficiens* strain USDA110 and *Bradyrhizobium* sp. strain ORS285.

## Results

### The *Bradyrhizobium diazoefficiens* USDA110 blr7537-encoded protein is a BclA homologue

Similar to other analyzed *Bradyrhizobium* strains^9^, no *bacA* homologous gene was identified in the genome of *B. diazoefficiens* USDA110. However, the *B. diazoefficiens* gene blr7537 was identified as a homolog of the *bclA* genes of *Bradyrhizobium* spp. ORS285 and ORS278^9^. The encoded protein has 70–72% identity and 80–81% similarity to the BclA proteins of strains ORS285 and ORS278. The protein has the same structure with an N-terminal SbmA-BacA transmembrane domain and a C-terminal ATPase domain. It thus encodes a potentially functional ABC transporter. Moreover, the genes in the 3 *Bradyrhizobium* species are located in syntenic regions that extend to over 200 kb. Therefore we designated blr7537 as *bclA*. Similarly as in strains ORS285 and ORS278, the *B. diazoefficiens* locus lacks genes that potentially encode additional components of the ABC transporter, such as a periplasmic binding protein for substrate binding and delivery to the transporter.

### *Bradyrhizobium diazoefficiens* USDA110 BclA is an NCR peptide transporter and functional in symbiosis

To test the *in vitro* and *in vivo* activity of the *B. diazoefficiens* USDA110 BclA protein, a deletion mutant of the *bclA* gene was constructed. The gene was also cloned into the plasmids pMG103 and pRF771, downstream of the *trp* promoter, and introduced into the *bclA*, *bacA* and *sbmA* mutants of *Bradyrhizobium* sp. ORS285, *S. meliloti* strain Sm1021 and *E. coli* strain BW25113, respectively.

**Figure 1:**
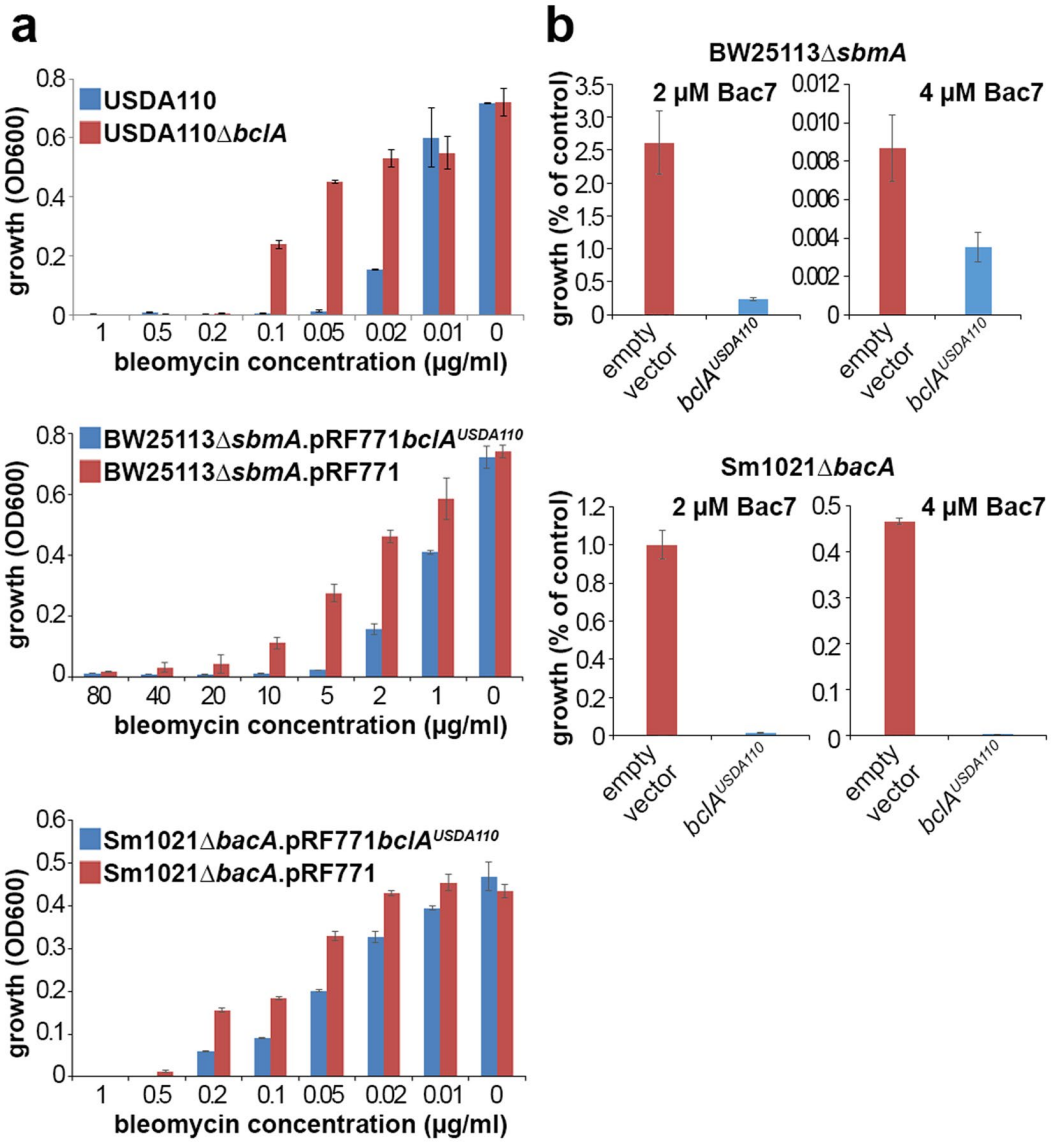
The USDA110 *bclA* gene confers sensitivity to the antibiotic bleomycin and the antimicrobial peptide Bac7. (**a**) Bleomycin sensitivity in *B. diazoefficiens* strain USDA110 and its *bclA* mutant derivative and in *E. coli* strain BW25113Δ*sbmA* and *S. meliloti* strain Sm1021Δ*bacA* carrying an empty vector or a vector expressing USDA110 *bclA*. Blue bars are for strains expressing *bclA* and red bars are for strains lacking the gene. Bleomycin concentrations were applied as indicated and growth was determined by optical density measurement at 600 nm with a plate reader after 72 h, 48 h or 24 h incubation for USDA110, Sm1021 and BW25113 respectively. (**b**) Survival of *E. coli* strain BW25113Δ*sbmA* (top) and *S. meliloti* strain Sm1021Δ*bacA* (bottom) derivatives carrying the empty pRF771 plasmid or the USDA110 *bclA* gene located on plasmid pRF771 after treatment with the peptide Bac7 at the indicated concentration. The surviving bacteria were counted and expressed as % from the water control treatment. E﻿rror bars in all panels are standard deviations.

Bleomycin and Bac7 are antimicrobial compounds which have intracellular targets, DNA and the ribosomes respectively, and they require active transport to be taken up in the bacterial cells. In *E. coli*, *S. meliloti* and *Bradyrhizobium* spp. ORS285 and ORS278, the uptake is mediated by the SbmA/BacA/BclA transporters^9, 17, 30^. Thus strains expressing one of these transporters display a significantly increased sensitivity to bleomycin or Bac7. We find that wild type *B. diazoefficiens* strain USDA110 or the *E. coli sbmA* and *S. meliloti bacA* mutants expressing USDA110 *bclA* from the pRF771 plasmid are more sensitive to bleomycin or Bac7 than the corresponding strains lacking *bclA* (Fig. 1), in agreement with BclA of USDA110 being able to transport these peptides.

**Figure 2:**
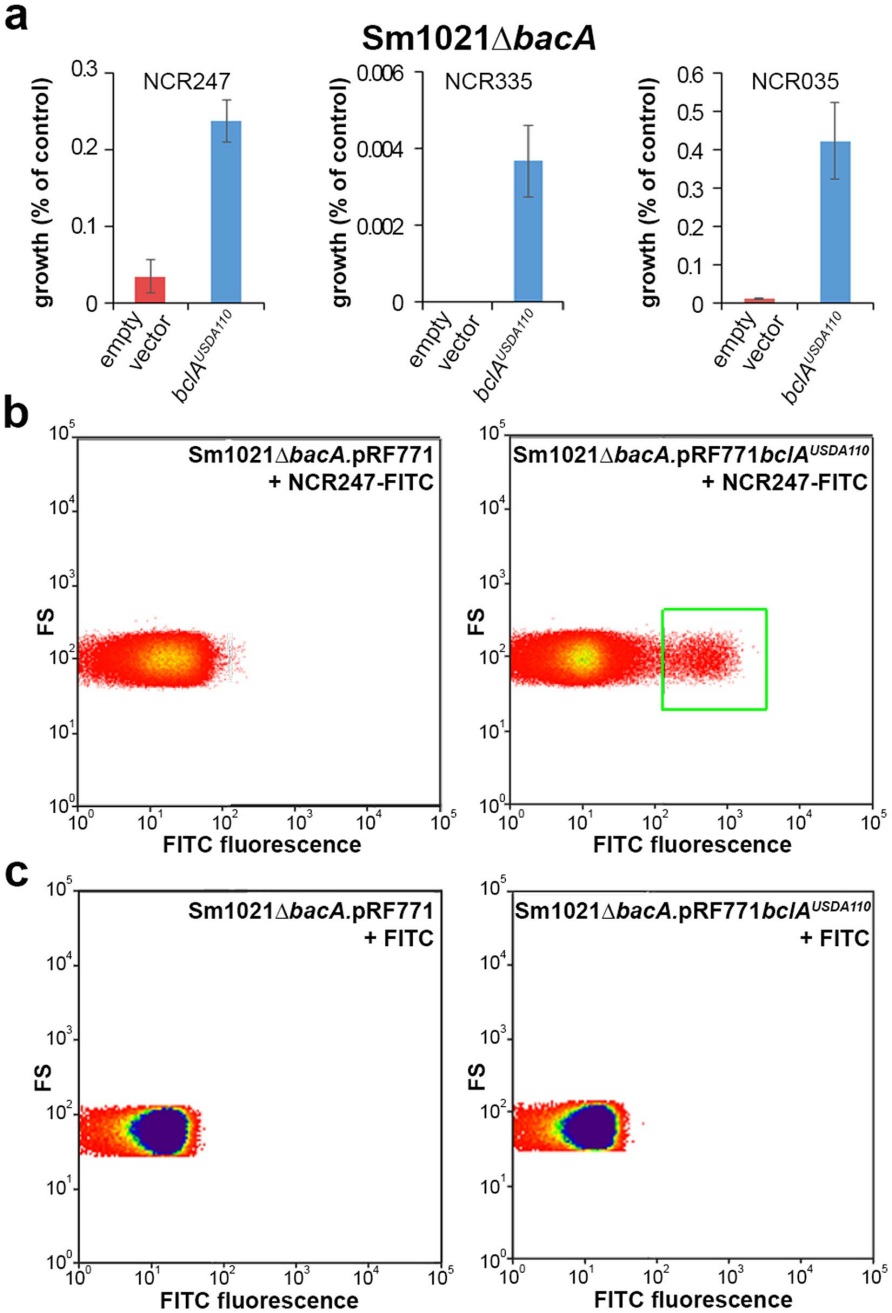
The USDA110 *bclA* gene confers resistance to antimicrobial NCR peptides and mediates the uptake of FITC-NCR247. (**a**) *S. meliloti* strain Sm1021Δ*bacA* derivatives expressing no *bacA*-related gene (empty vector) or the USDA110 *bclA* gene were incubated with NCR247, NCR335 or NCR035 or with water (control) and the surviving bacteria were counted and expressed as % from the control treatment. Error bars in all panels are standard deviations. (**b**) FITC-NCR247 uptake by *S. meliloti* strain Sm1021Δ*bacA* or its derivative expressing the USDA110 *bclA* gene was measured by flow cytometry in the presence of trypan blue to quench extracellular fluorescence. FITC-positive cells are marked with a green box. (**c**) FITC is not taken up by *S. meliloti* derivatives. FITC-treated bacteria were analyzed by flow cytometry in the presence of trypan blue to quench extracellular fluorescence. No FITC-positive cells were detected.

Contrary to bleomycin and Bac7, the sensitivity to antimicrobial NCR peptides is reduced in the presence of *sbmA*, *bacA* or *bclA* ^8, 9^. This opposite response is likely because the toxicity of the NCR peptides resides in their potential to provoke membrane permeability and loss of membrane potential, rather than in the inhibition of some intracellular process. Also the *bclA* gene of USDA110 is able to reduce the sensitivity to NCR peptides in the *S. meliloti bacA* mutant (Fig. 2a). In addition, similarly as shown before for the *S. meliloti bacA* or *Bradyrhizobium* strain ORS285 *bclA* genes^9^, the expression of USDA110 *bclA* promotes the uptake of an FITC-modified NCR247 peptide into the *S. meliloti* strain Sm1021Δ*bacA* (Fig. 2b), while FITC alone is not taken up (Fig. 2c).

**Figure 3:**
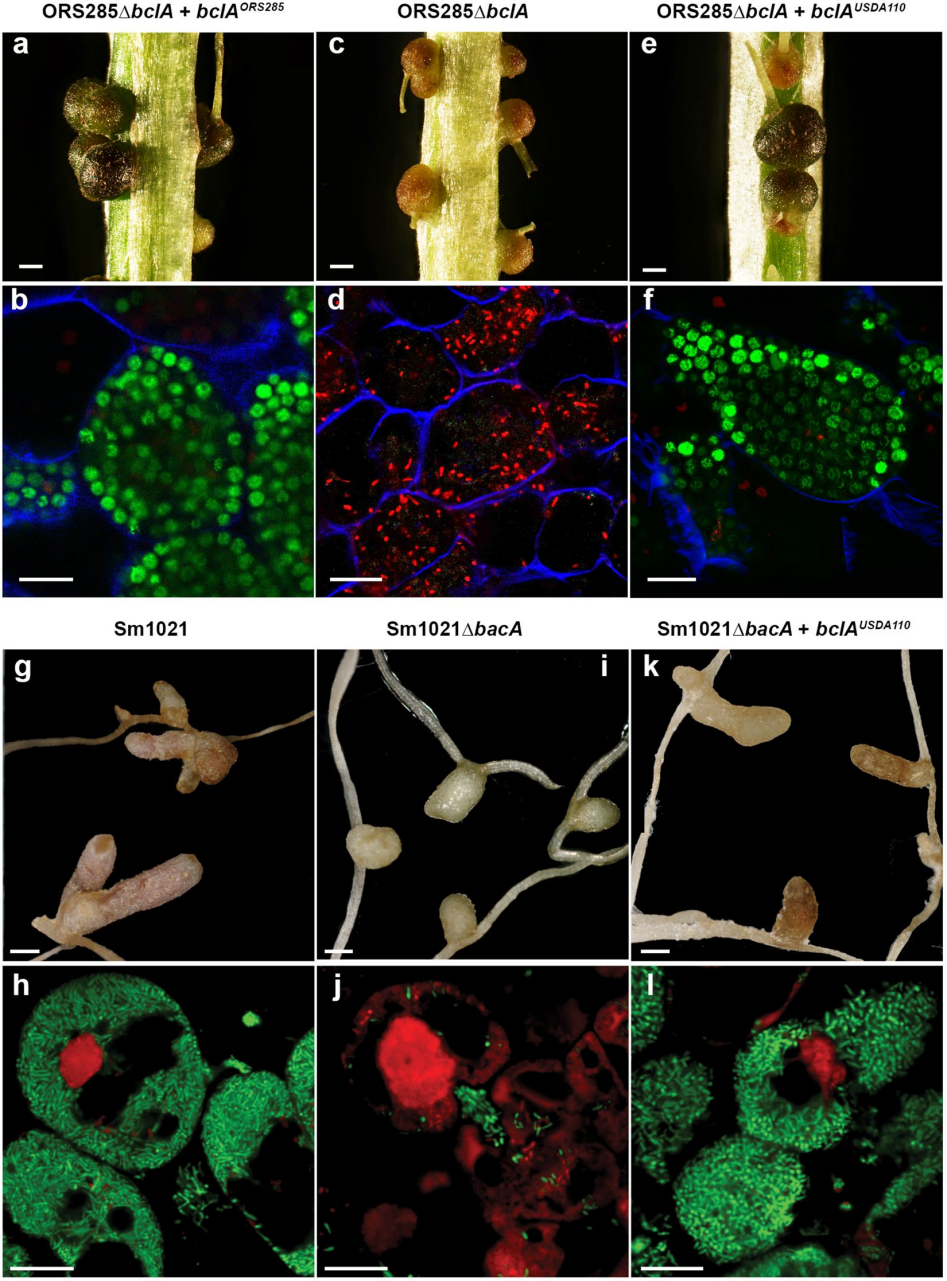
The *bclA* gene of *B. diazoefficiens* USDA110 complements the *bclA* mutation in *Bradyrhizobium* sp. ORS285﻿Δ*bclA﻿* and the *bacA* mutation in *S. meliloti* Sm1021Δ*bacA*. (**a**,**c**,**e**) Phenotype of *A. indica* nodules at 14 dpi, infected with the indicated strains. Scale bars are 1 mm. (**b**,**d**,**f**) Bacteroid viability determined by live/dead and calcofluor white staining of nodule sections and confocal microscopy in *A. indica* nodules induced by the indicated strains. Scale bars are 10 µm. (**g**,**i**,**k**) Phenotype of *M. sativa* nodules at 28 dpi, infected with the indicated strains. Scale bars are 1 mm. (**h**,**j**,**l**) Bacteroid viability determined by live/dead staining of nodule sections and confocal microscopy in *M. sativa* nodules induced by the indicated strains. Scale bars are 10 µm.

Finally, the *bclA* gene of USDA110 can complement the ORS285Δ*bclA* mutant for bacteroid differentiation in *A. indica* nodules (Fig. 3a–f). This mutant induces small nodules in which bacteria do not differentiate and die as revealed by live/dead staining of nodule sections^9^ (Fig. 3c,d). The USDA110 *bclA* gene, similarly as the ORS285 *bclA* gene, and introduced into this mutant on the pMG103 plasmid, restores the wild type phenotype with the formation of large nodules inhabited with well-formed spherical bacteroids that remain alive (Fig. 3a,b,e,f). Plants inoculated with both complemented strains showed vigorous growth, contrary to those inoculated with the un-complemented mutant indicating that nitrogen fixation defect of the mutant is restored by the USDA110 *bclA* gene. Moreover, the USDA110 *bclA* gene also complements in part the Sm1021Δ*bacA* strain for nodulation of *M. sativa* (Fig. 3g–l). The nodules induced by the complemented strain become elongated and pinkish, compared to the small white nodules induced by the *bacA* mutant (Fig. 3g,i,k). The *bacA* mutant bacteria die as soon as they are released inside the nodule cells and exposed to the NCR peptides^8^(Fig. 3j). The bacteroids of the complemented strain however are viable within the symbiotic cells indicating that the hypersensitivity of the *bacA* mutant to the NCR peptides is suppressed by the *bclA* gene of USDA110 (Fig. 3l). Nevertheless, the defect of the *S. meliloti bacA* mutation is only partially repaired by *bclA* of USDA110 because nodules do not fix nitrogen and do not support plant growth (data not shown). This phenotype is similar to the one obtained with the *S. meliloti bacA* complementation by *bclA* of strain ORS285^9^ or even by more similar *bacA* genes of *Sinorhizobium* or *Rhizobium* species^31^. This suggests that although rhizobial *bacA* and *bclA* genes have overlapping specificity for peptide uptake, they may also display differences in the set and/or amount of NCR peptides they can handle, probably because they evolved in the context of specific interactions with host plants, each producing its specific arsenal of NCR peptides. Nevertheless, the USDA110 *bclA* gene seems to be capable to treat the *Aeschynomene* NCR peptides.

Together, the bleomycin, Bac7 and NCR peptide sensitivity assays as well as the *in vivo* complementation of the *Bradyrhizobium* sp. ORS285 *bclA* mutantion or the *S. meliloti bacA* mutation indicate that the *bclA* gene of *B. diazoefficiens* strain USDA110 is functional and has a similar activity as the *bclA* gene of *Bradyrhizobium* strain ORS285 and the *S. meliloti bacA* gene.

### *Bradyrhizobium diazoefficiens* USDA110 BclA is not required for symbiosis with NCR-lacking soybean

**Figure 4:**
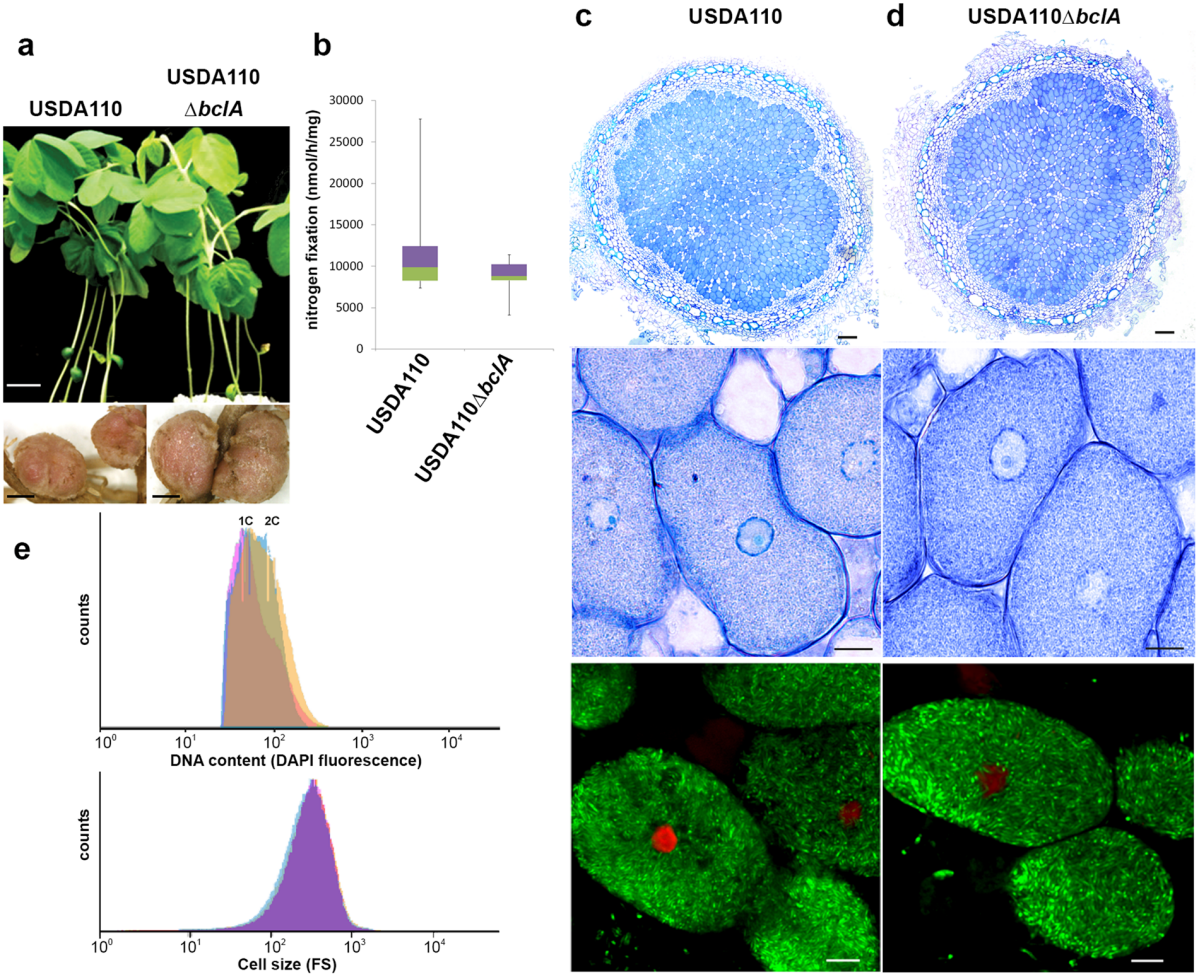
The *B. diazoefficiens bclA* mutant is not affected in symbiosis with soybean. (**a**) Plant growth and nodule phenotype of *G. max* plants inoculated with *B. diazoefficiens* USDA110 wild type and the *bclA* mutant at 14 dpi. Scale bars are 3.5 cm (top panels) and 1 mm (bottom panels). (**b**) The nitrogen fixation activity of *G. max* plants inoculated with *B. diazoefficiens* USDA110 wild type and the *bclA* mutant, measured by the acetylene reduction assay per mg of fresh nodule weight (nmol ethylene produced per hour incubation and per mg nodule weight). Box-plots represent in the rectangle the first quartile to the third quartile, divided by the median value, whiskers above and below the box show the minimum and maximum measured values. (**c**,**d**) Toluidine blue stained thin sections (two top rows) and confocal microscopy of bacteroid viability determination by live/dead staining (bottom row) of *G. max* nodules induced by USDA110 or USDA110Δ*bclA*. Scale bars are 100 µm for the top panels and 10 µm for the other panels. (**e**) Flow cytometry analysis of DNA content by DAPI fluorescence (top) and of cell size by forward scatter (bottom) in free-living *B. diazoefficiens* USDA110 (pink) or bacteroids isolated from *G. max* nodules infected with USDA110 wild type (blue) or USDA110Δ*bclA* (orange). The forward scatter and DAPI fluorescence profiles are completely overlapping for the 3 samples.

In agreement with the taxonomic position of soybean within the Millettioids, the *B. diazoefficiens* strain USDA110 bacteroids in soybean nodules are undifferentiated and display no cell enlargement and polyploidy as revealed by microscopy of nodule sections (Fig. 4c) and flow cytometry analysis of purified bacteroids (Fig. 4e). We tested whether the *bclA* gene in strain USDA110 is required for symbiosis with soybean. The *bclA* mutant of USDA110 was undistinguishable from the wild type strain for all parameters analyzed, including nodule tissue structure and bacterial occupation, bacterial viability, morphology, size and DNA content as well as nitrogen fixation (Fig. 4a–e). Thus the *Bradyrhizobium bclA* gene, similarly to *bacA* in other rhizobium species, is not required for symbiosis when bacteroids are not constrained by the host plant-produced NCR peptides to differentiate into an elongated and polyploid morphotype.

### *Bradyrhizobium diazoefficiens* USDA110 BclA is also not required for symbiosis with NCR-producing *Aeschynomene afraspera*

**Figure 5:**
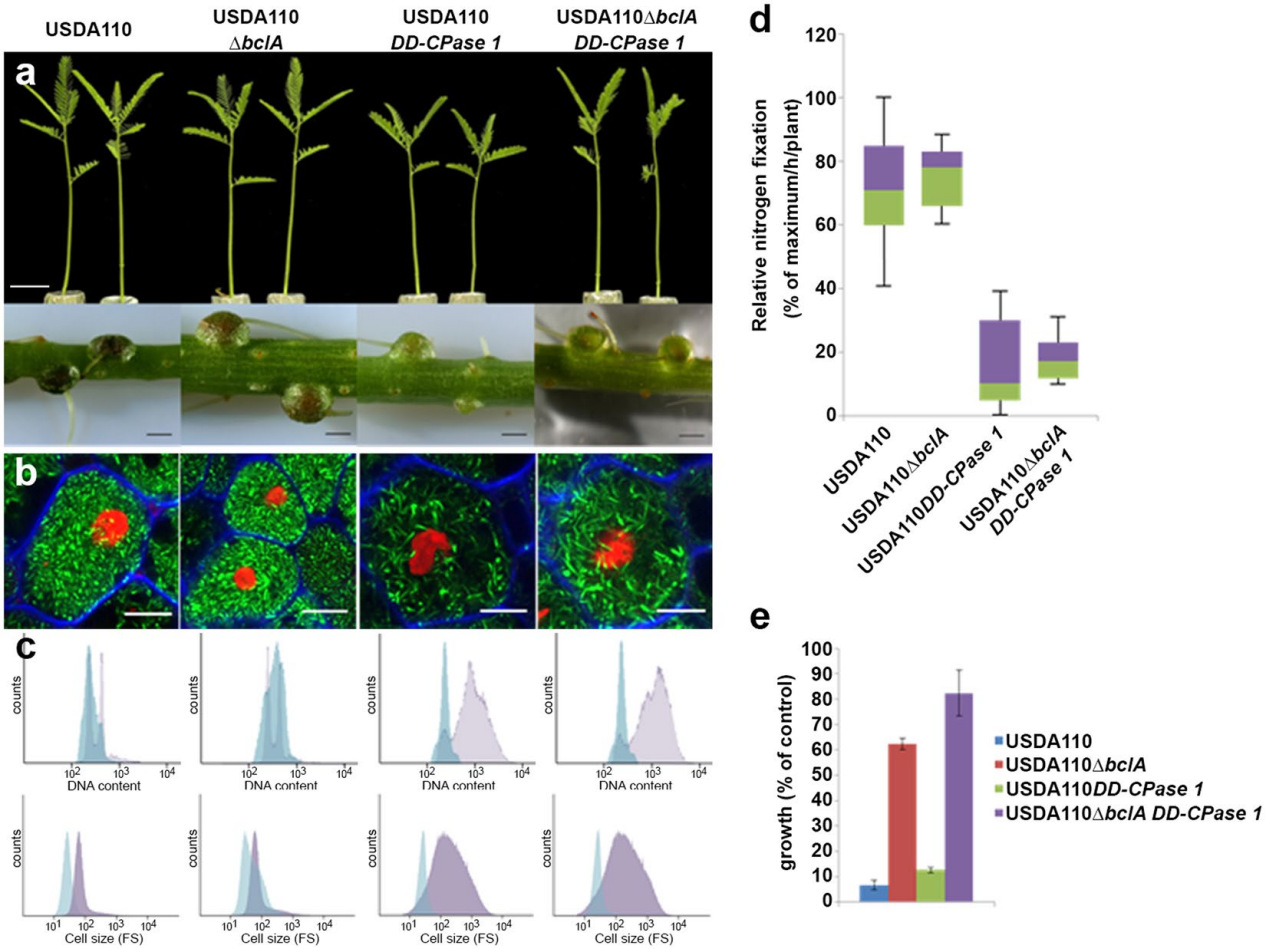
The *B. diazoefficiens bclA*, *DD-CPase1*, *bclA/DD-CPase1* mutant phenotypes in symbiosis with *A. afraspera*. (**a**) Plant growth and nodule phenotype of *A. afraspera* plants inoculated with *B. diazoefficiens* USDA110 wild type and indicated mutants at 14 dpi. Scale bars are 2 cm (top panels) and 1 mm (bottom panels). (**b**) Confocal microscopy of bacteroid viability determination by live/dead and calcofluor white staining of *A. afraspera* nodules induced by USDA110 wild type and indicated mutants. Scale bars are 10 µm. (**c**) Flow cytometry analysis of DNA content by DAPI fluorescence and of cell size by forward scatter (FS) in free living *B. diazoefficiens* USDA110 (blue) or bacteroids isolated from *A. afraspera* nodules infected with USDA110 wild type and indicated mutants (pink). (**d**) Relative nitrogen fixation activity (% of maximum/h/plant) of *A. afraspera* plants inoculated with *B. diazoefficiens* USDA110 wild type and indicted mutants, measured by the acetylene reduction assay per plant. Box-plots represent in the rectangle the first quartile to the third quartile, divided by the median value, whiskers above and below the box show the minimum and maximum measured values. (**e**) Bleomycin sensitivity in *B. diazoefficiens* strain USDA110 and its *bclA, DD-CPase1*, *bclA/DD-CPase1* mutant derivatives at 0,0375 µg bleomycin/ml. Growth was determined by optical density measurement at 600 nm with a plate reader after 72 h. Error bars in panels (d) and (e) are standard deviations.

*B. diazoefficiens* strain USDA110, which is a natural soybean symbiont, can also form functional nodules on *A. afraspera* ^29, 32^. The strain forms nodules composed of a central zone with fully infected symbiotic cells and a cortex layer surrounding the infected cells. This nodule organization is very similar to the histology of nodules induced by the natural *Aeschynomene* symbiont *Bradyrhizobium* strain ORS285 (Fig. S1). Nevertheless, USDA110 is a less efficient nitrogen fixer, supporting lower plant biomass production and nitrogen accumulation than ORS285^32^ (Fig. S2). The latter strain forms elongated and polyploid bacteroids on *A. afraspera* and requires the *bclA* gene for elongated bacteroid formation^9^. Therefore, we analyzed the bacteroid type formed by strain USDA110 within *A. afraspera* nodules and the role of the USDA110 *bclA* gene. Unexpectedly, observations by confocal microscopy showed that USDA110 bacteroids in *A. afraspera* nodules were not or only very slightly elongated (Fig. 5b), contrary to bacteroids of strain ORS285 which are strongly elongated^4, 9^. To confirm this unpredicted observation we used flow cytometry analysis of the bacteroid cell size, determined by the forward scatter (a measure for cell size), and the DNA content, measured by DAPI fluorescence. A slight increase in size of the bacteroids compared to the bacteria in culture was measured but this was not accompanied with an increase in the DNA content of the bacteroids (Fig. 5c). Thus the USDA110 bacteroids are much less or hardly differentiated compared to ORS285 bacteroids which have, besides the strong cell enlargement, also a marked increase in DNA content^4, 9^. The absence of a pronounced differentiation of USDA110 bacteroids is not likely resulting from a defect in *NCR* gene expression in USDA110-infected nodules since five tested *NCR* genes were expressed at similar or even higher levels in USDA110-infected nodules compared to ORS285-infected nodules (Fig. S3).

In agreement with the absence of differentiation of the wild type USDA110, the USDA110Δ*bclA* mutant was not affected in symbiosis with *A. afraspera*: nodules infected with wild type or mutant were similar, supported plant growth and fixed nitrogen to the same extent (Fig. 5a,d), both types of nodules contained symbiotic cells which were completely infected with bacteroids that seemed not or only slightly elongated (Fig. 5b; Fig. S1) which was confirmed by flow cytometry (Fig. 5c).

### BclA is not required for the formation of differentiated bacteroids in *Aeschynomene afraspera* nodules by the USDA110 *DD-CPase1* mutant

Contrary to the wild type USDA110 strain, the UDSA110 *DD-CPase1* mutant forms strongly elongated and polyploid bacteroids in the *A. afraspera* nodules^29^ (Fig. 5b,c), indicating that by affecting their cell wall strength, the bacteria become sensitive to the NCR differentiation signals produced by the nodule cells. Nevertheless, the nitrogenase activity of plants infected with the UDSA110 *DD-CPase1* mutant is strongly reduced, in large part because the mutant induces much less nodules than the wild type^29^ (Fig. 5d). We created a *bclA*/*DD-CPAse1* double mutant to determine whether the cell wall-determined bacteroid differentiation is depending on the BclA peptide transporter. Unexpectedly, the double mutant exhibited a similar symbiotic phenotype than the *DD-CPase1* single mutant (Fig. 5b–d). This result indicates that the differentiation of USDA110, made possible by the inactivation of the *DD-CPase1* gene, is independent of BclA.

A possible explanation could be that the *DD-CPase1* mutation increases the permeability of cells for peptides, including NCR peptides, rendering the BclA transporter superfluous. To test this possibility, we measured sensitivity of strains against the peptide bleomycin which needs to be internalized to target the bacterial DNA. We found that the *DD-CPase1* mutant strain displays a sensitivity to bleomycin similar to the wild type strain (Fig. 5e), indicating that the peptidoglycan remodeling, induced by the mutation, does not interfere with peptide uptake. Similarly, the double mutant is just as much or even slightly more resistant to bleomycin than the *bclA* mutant (Fig. 5e).

### BclA and DD-CPase1 in *Bradyrhizobium* strain ORS285 act independently in bacteroid differentiation

**Figure 6:**
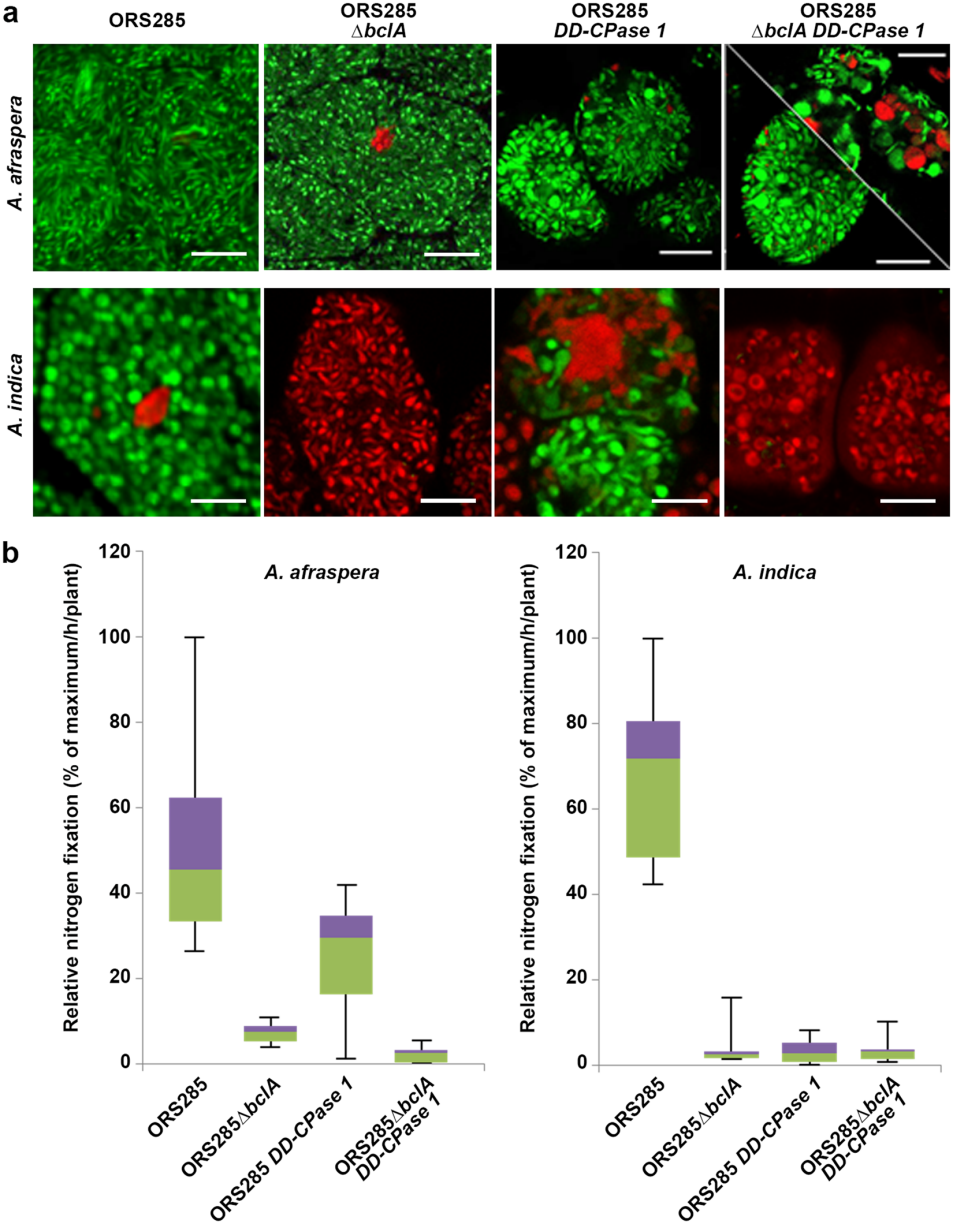
The *Bradyrhizobium* strain ORS285 *ΔbclA*, *DD-CPase1*, *ΔbclA/DD-CPase1* mutant phenotypes in symbiosis with *A. afraspera* and *A. indica*. (**a**) Confocal microscopy of bacteroid viability determination by live/dead staining of *A. afraspera* and *A. indica* nodules induced by ORS285 wild type and the indicated mutants. Scale bars are 10 µm. (**b**) Relative nitrogen fixation activity (% of maximum/h/plant) of *A. afraspera* and *A. indica* plants inoculated with ORS285 wild type and the indicted mutants, measured by the acetylene reduction assay per plant. Box-plots represent in the rectangle the first quartile to the third quartile, divided by the median value, whiskers above and below the box show the minimum and maximum measured values.

To further explore the interdependence of BclA and DD-CPase1 in bacteroid differentiation, we created the double mutant also in *Bradyrhizobium* strain ORS285. This strain forms nodules on *A. afraspera* as well as on *A. indica* in which it differentiates into either elongated polyploid or spherical polyploid bacteroids respectively^4^. A *bclA* mutation in this strain blocks the differentiation process in both hosts^9^ (Fig. 6a) while a *DD-CPase* mutation induces hypertrophied bacteroids^29^ (Fig. 6a). In a similar way as in USDA110, the ORS285 double mutant was still capable to induce strongly enlarged bacteroids in nodules of both *A. afraspera* and *A. indica* (Fig. 6a). In *A. indica*, the *bclA* mutation induced death of the bacteria as revealed by the red fluorescence in the live/dead staining procedure of nodule sections (Fig. 6a). Even if in the double mutant, many bacteroids were strongly enlarged (Fig. 6a), others remained undifferentiated. This may be related to bacterial death induced by the *bclA* mutation in such a way that many bacteria die before having the chance to enlarge. Thus, these results indicate that the *bclA* and *DD-CPase1* mutations have a cumulative effect and that the *bclA* function is not upstream of the bacterial differentiation provoked by the *DD-CPase1* mutation. The cumulative effect of the two mutations is also observed when measuring with the acetylene reduction assay the nitrogenase activity of *A. afraspera* nodules (Fig. 6b). The *bclA* mutation has a stronger impact on nitrogen fixation than the *DD-CPase1* mutation and the double mutant has the same low nitrogenase activity as the *bclA* single mutant while on the other hand bacteroids of the double mutant resemble morphologically more the *DD-CPase1* bacteroids.

## Discussion

The BacA transporter is widespread but not universal in bacteria. It has been recruited by rhizobia that infect host plants producing NCR peptides in their infected nodule cells. In such host cells, the BacA protein is essential for the rhizobia to differentiate and establish a chronic infection. However, BacA is not critical for symbiosis in host cells of legumes that do not produce NCR peptides^8^. Rhizobia such as *Bradyrhizobium* spp., which lack a *bona fide bacA* gene but infect legume hosts that do produce NCR peptides in the infected nodule cells, use the different but related transporter BclA^9^. Here we show that the BclA function in *B. diazoefficiens*, in analogy with the BacA function in other rhizobia, is superfluous for chronic infection of soybean symbiotic cells that do not produce NCR peptides or when the bacterial strain is inherently non-responsive to the peptides produced by *A. afraspera* symbiotic cells. In agreement with our current findings, we previously demonstrated that the *bacA* gene of the broad host range bacterium *Sinorhizobium fredii* strain HH103 is not required for symbiosis with soybean^26^. These results, together with previously published studies support the view that the major, if not sole symbiotic role of SbmA_BacA domain transporters is the import of NCR peptides.

*B. diazoefficiens* strain USDA110 is not a natural symbiont of *Aeschynomene* host plants. The strain produces the same major Nod factors as the genuine *Aeschynomene* symbiont *Bradyrhizobium* strain ORS285 and is able to induce with equal efficiency nodules on *A. afraspera* roots which have a normal histological organization but a reduced nitrogen fixation and plant growth promoting activity^32^ (Fig. S2). However a striking difference between the two strains is the absence of marked differentiation of the USDA110 bacteroids (this work) while the ORS285 bacteroids strongly elongate and become polyploid^4^. The absence of differentiation of *B. diazoefficiens* strain USDA110 in *A. afraspera* nodules is unlikely to be the result of its *bclA* gene being a defective allele because we found that the USDA110 *bclA* mutant has a similar response to bleomycin as the corresponding mutants in strains ORS285 and ORS278. The USDA110 *bclA* gene fully complements the symbiotic defect of the ORS285 *bclA* mutation for bacteroid differentiation in *Aeschynomene*. It also complements *S. meliloti bacA* and *E. coli sbmA* mutants, similar to the corresponding genes of ORS285 and ORS278^9^, for Bac7- and NCR-uptake and -sensitivity and for symbiosis with *M. sativa*. Together, these findings demonstrate that USDA110 BclA has the same capacity as BclA from ORS285 or BacA from *S. meliloti* to transport a broad range of antimicrobial peptides, including Bac7, bleomycin and NCR peptides. They also tell us that USDA110 BclA does not lack the ability to uptake some specific peptide or set of peptides produced in *Aeschynomene* nodules which would explain the inability of the strain to differentiate within these nodules. Moreover, transcriptome analysis by RNA-seq (data not shown) revealed that *bclA* of USDA110 is weakly expressed, in culture as well as in soybean or *A. afraspera* nodules, but the orthologous gene of ORS285 is similarly weakly expressed. It is also unlikely that the lack of differentiation of USDA110 bacteroids results from a defect in NCR gene expression in USDA110-infected nodules since five tested NCR genes were expressed at similar or even higher levels in USDA110-infected nodules compared to ORS285-infected nodules.

A candidate factor for the strain USDA110 recalcitrance to bacteroid differentiation in *A. afraspera* nodules is the production of hopanoids^33^. Hopanoids are a bacterial class of lipids, produced by certain bacteria, notably the bradyrhizobia but not the other rhizobal genera. These lipids, which are similar to eukaryotic steroid lipids, render membranes more rigid and resistant to membrane stresses, including to antimicrobial peptides^34, 35^. Strain USDA110 is remarkable among the bradyrhizobia because of its very high accumulation of hopanoids, up to 40% of the total cellular lipid fraction^33^. The amount of hopanoids accumulating in the membranes of strain ORS285 is currently unknown but it could be sufficiently low for NCR responsiveness while the high hopanoid content in USDA110 could make this strain insensitive to the NCR signals of the host. In agreement with this hypothesis, an *hpnH* mutant of USDA110, affected in the synthesis of a specific type of hopanoids, the C35 hopanoids, is drastically impaired in symbiosis with *A. afraspera* but not with soybean; the mutant, in contrast to the wild type, is also sensitive to the antimicrobial activity of NCR peptides^36^. Moreover, even if the *Bradyrhizobium* USDA110 and ORS285 strains have a high proportion of shared genes, they have nevertheless also a substantial number of unique genes and they belong to phylogenetically distinct clades in the *Bradyrhizobium* genus^37, 38^. A recent comparative genome analysis of ten *Bradyrhizobium* strains, including strains USDA110 and ORS285, revealed that USDA110 has over 3000 unique genes while ORS285 had almost 500 genes not shared with the other strains included in the study^38^. Probably the different genetic repertoires of the USDA110 and ORS285 strains contribute to their differential response to the bacteroid differentiation factors produced by the symbiotic host cells. This difference could be exploited to identify new bacterial factors involved in the differentiation process and the response to NCR peptides. Whatever its molecular basis, the weak or non-responsiveness of strain USDA110 to the host-produced bacteroid differentiation factors most likely reflects the fact that this strain has co-evolved with NCR-lacking soybean and not with NCR-producing *Aeschynomene* or other NCR-producing hosts.

It is interesting to note that although the root nodules infected with the strain USDA110 fix nitrogen, they do so less efficiently when compared to nodules infected with strain ORS285; they supported less plant growth with the accumulation of lower amounts of nitrogen in the plant biomass, indicating that the undifferentiated bacteroids of USDA110 are less efficient than the differentiated bacteroids of ORS285. We recently demonstrated an analogous correlation between the level of bacteroid differentiation and symbiotic performance in the interactions of two *M. truncatula* accessions with four different *Sinorhizobium* strains^39^. These concordant observations in different legumes suggest that the differentiation of bacteroids may contribute - by a mechanism that still has to be clarified - to the nitrogen fixation efficiency of rhizobium-legume symbioses^40–42^. Variability in the response to NCR peptides can be at the basis of such variations in nitrogen fixation efficiency of *Rhizobium* strains. This has been nicely illustrated recently in the *S. meliloti*-*M. truncatula* interaction^43, 44^. Allelic variations in single NCR genes between *M. truncatula* accessions can determine the nitrogen fixation effectiveness of a particular *S. meliloti* strain while not affecting other strains. Thus the genome of rhizobial symbionts should be optimally aligned with the spectrum of NCR peptides produced by the nodule cells for efficient bacteroid differentiation and nitrogen fixation.

The BclA/BacA transporters provide protection against the antimicrobial activity of NCR peptides, both *in vitro* and *in vivo*, and in addition, the transporters are also required for bacterial elongation and polyploidization^8, 9^ (this work). This implies two possibilities concerning the molecular function of these transporters: either their sole function is providing protection against the antimicrobial stress in the nodule cells and the lack of bacteroid differentiation in the corresponding mutants is an indirect consequence of the reduced fitness of these mutants; alternatively, the transporters are, in addition to providing protection, also directly involved in the activation of the differentiation mechanism leading to cell enlargement and polyploidization, for example by transporting the NCR peptide signals into the bacterial cytoplasm and so allowing the peptides to interfere with the bacterial cell cycle machinery and other intracellular targets^6, 7^. Our phenotypic analyses of the *bclA*/*DD-CPase1* double mutants in the *Bradyrhizobium* strains USDA110 and ORS285 are in favor of the first option; they suggest that the sole role of BclA is the protection against the NCR stress. The abnormal and excessive differentiation provoked by the *DD-CPase1* mutation uncovered the differentiation potential of the *bclA* mutants. This interpretation of our data is in agreement with our previous observation that *in vitro* the *bacA* mutant of *S. meliloti* responds similarly as the wild type strain to NCR treatment by elongation and DNA amplification at peptide concentrations below their toxic level^8^. This implies that the NCR peptides are perceived at the outer or inner membrane, activating a signaling process that induces the differentiation process or alternatively, that NCR peptides can enter in a BclA/BacA independent manner the bacterial cells to reach their cytosolic targets for differentiation.

## Methods

### Bacterial growth conditions

*Bradyrhizobium* spp. strains were grown in yeast mannitol^45^ (YM) or buffered nodulation medium for bacteria (BNM-B)^9^ at 30 °C, *E. coli* and *S. meliloti* strains in Luria-Bertani (LB) medium at 37 °C or 30 °C respectively. Antibiotics for strain and plasmid selection were used at the following concentrations: streptomycin (500 µg/ml); spectinomycin (100 µg/ml); chloramphenicol (12.5 µg/ml); gentamycin (15 or 50 µg/ml); carbenicilin (50 µg/ml); tetracycline (10 µg/ml); kanamycin (50 or 200 µg/ml); neomycin (120 µg/ml); and nalidixic acid (25 µg/ml).

### Construction of bacterial mutants and complemented strains

Standard molecular biology techniques were used for all cloning work. The construction of the *B. diazoefficiens* USDA110 *bclA* deletion mutant (USDA110*ΔbclA*) was performed with the same strategy as the previously described mutant in *Bradyrhizobium* strain ORS285^9^. The following primers were used for the amplification of the upstream and downstream region of the *bclA* gene: GATAGAAAGCTTAAGCGTCCGGTGGTCACCGTCACCTG (forward, upstream region); GTCTTGCGCCGGATCCAGAGCTCTGTCTCCTGAGGGGATG (reverse, upstream region); ACAGAGCTCTGGATCCGGCGCAAGACGATCGCTATCGTAG (forward, downstream region); GATAGAACTAGTGTTCGGCACCTCGGACGCCTTCTAC (reverse, downstream region).

The mutant strains ORS285*ΔbclA*, ORS285*ΔDD-CPase1* and USDA110*ΔDD-CPase1* were obtained from previous studies^9, 29^. The double mutants ORS285*ΔbclADD-CPase1* and USDA110 *ΔbclADD-CPase1* were constructed from the corresponding single mutant in *bclA* and using the respective *DD-CPase1* pVO155-npt2-GFP mutagenesis plasmids which were introduced by triparental mating, as described^29^.

The *bclA* gene of *B. diazoefficiens* USDA110 was amplified by PCR using the following primer couple: TTATCGTCTAGACCCTCAGGAGACAGAGCTCTGTGAAG (forward) and CATGATGGATCCGATCGTCTTGCGCCTCAGCGCGCCACGGTC (reverse). The PCR fragment was cloned into the *Xba*I and *Bam*HI sites of plasmid pRF771^46^. The resulting plasmid was introduced into *S. meliloti* Sm1021Δ*bacA* by tri-parental conjugation and into *E. coli* BW25113Δ*sbmA* by electroporation. The same USDA110 *bclA*-encoding PCR fragment was also introduced in plasmid pMG103^47^. First, the *trp* promoter was amplified with primers GTGCCGAATTCGGCAAATATACTGAAATAGGTGTTG (forward) and GAGTGCATGCGGTACCGGATCCATGGAATCTAGATTTAAAGTACTTCGAA (reverse) and introduced into the *Eco*RI and *Sph*I sites of pMG103. The *bclA* PCR product was subsequently cloned in the *Xba*I and *Bam*HI sites of this pMG103 derivative. The resulting construct was introduced into *Bradyrhizobium* sp. ORS285Δ*bclA* by electroporation.

### Peptide sensitivity assays, single-cell NCR peptide uptake assay and flow cytometry

All sensitivity assays were as described before^9^. The peptide uptake assay by flow cytometry was as described^48^.

### Real-Time Quantitative PCR Expression Analyses

The relative expression level of *NCR* genes was determined by RT-qPCR using methods for RNA extraction, cDNA synthesis, quantitative PCR, normalization and with primers previously described^4^.

### Plant growth and nodulation

The soybean variety used in this study was *Glycine max* Williams 82. Seeds were cleaned with 100% ethanol for 30 sec and then surface-sterilized with bleach for 15 min. *A. indica* and *A. afraspera* seeds were surface sterilized using sulfuric acid (30 min) and bleach (30 min). Sterilized seeds were then transferred at 28 °C in the darkness on agar plates (0.8%) with tap water for germination. One-day old seedlings were transferred to test tubes containing liquid BNM^49^. Seedlings were grown at 28 °C with a 16 h light regime and 70% humidity. Seven days after germination, each seedling was inoculated with 1 ml of a bacterial suspension adjusted to an OD_600_ = 0.1.

### Symbiotic analyses

Nitrogen-fixation assays were performed with ten plants per condition at 14 days post-inoculation, as previously described^9^. Plant biomass production was determined from the fresh weight of shoots that were removed from whole plants. The nitrogen and carbon content was determined on pools of three de-nodulated whole plants that were dried in an oven at 60 °C for 48 h. The dried samples were analyzed with an Isoprime element analyzer (Elementar).

For microscopy analysis, nodules were harvested, embedded with agarose (6%), and then freshly sectioned with a Leica VT1200S vibratome (Leica Microsystems GmbH) into 50 µm tissue slices. Slices were incubated for 20 min in Live/Dead BacLight (Molecular Probes) staining solution in Tris buffer (50 mM), pH 7.0, containing 0.01% CalcofluorWhiteM2R (Sigma). Sections were washed to remove excess of dye and observed using a Leica TCS SP8x confocal microscope.

Toluidine blue staining of thin sections was made as follows. Nodules were fixed in 1% glutaraldehyde/4% paraformaldehyde. After washing, nodules were dehydrated in ethanol series and embedded in Technovit 7100 resin (KulzerHistoTechnik) according to the manufacturer’s instructions. Five µm sections were obtained with a Leica RM2155 microtome and stained with toluidine blue (0.005%). Bright field microscopy was performed with an Eclipse 80i microscope (Nikon).

### Bacteroid purification and flow cytometry analysis

Bacteroids were purified as described^24^. Bacteria were then fixed by heat treatment (70 °C, 10 min) and stained by DAPI (20 µg/ml) before flow cytometry analysis using a MoFlo ASTRIOS flow cytometer (Beckman Coulter). The bacterial DNA content was assessed by the DAPI fluorescence with a 355-nm laser line. Each single event was recorded and analyzed with the Summit 6.2 software (Beckman Coulter).

### Data Availability

All data generated or analyzed during this study are included in this published article (and its Supplementary Information files).

## Electronic supplementary material

Supplementary information
