# Supplementary material for: Integrated roles of BclA and DD-carboxypeptidase 1 in Bradyrhizobium differentiation within NCR-producing and NCR-lacking root nodules
Source: Sci Rep. 2017 Aug 22;7:9063. doi: 10.1038/s41598-017-08830-0 (PMC5567381; doi:10.1038/s41598-017-08830-0)
Supplement: Supplementary file 1 — Supplementary information [file 41598_2017_8830_MOESM1_ESM.pdf]

Integrated roles of BclA and DD-carboxypeptidase 1 in *Bradyrhizobium* differentiation within NCR-producing and NCR-lacking root nodules

Quentin Barrière, Ibtissem Guefrachi, Djamel Gully, Florian Lamouche, Olivier Pierre, Joël Fardoux, Clémence Chaintreuil, Benoît Alunni, Tatiana Timchenko, Eric Giraud, Peter Mergaert

Supplementary information

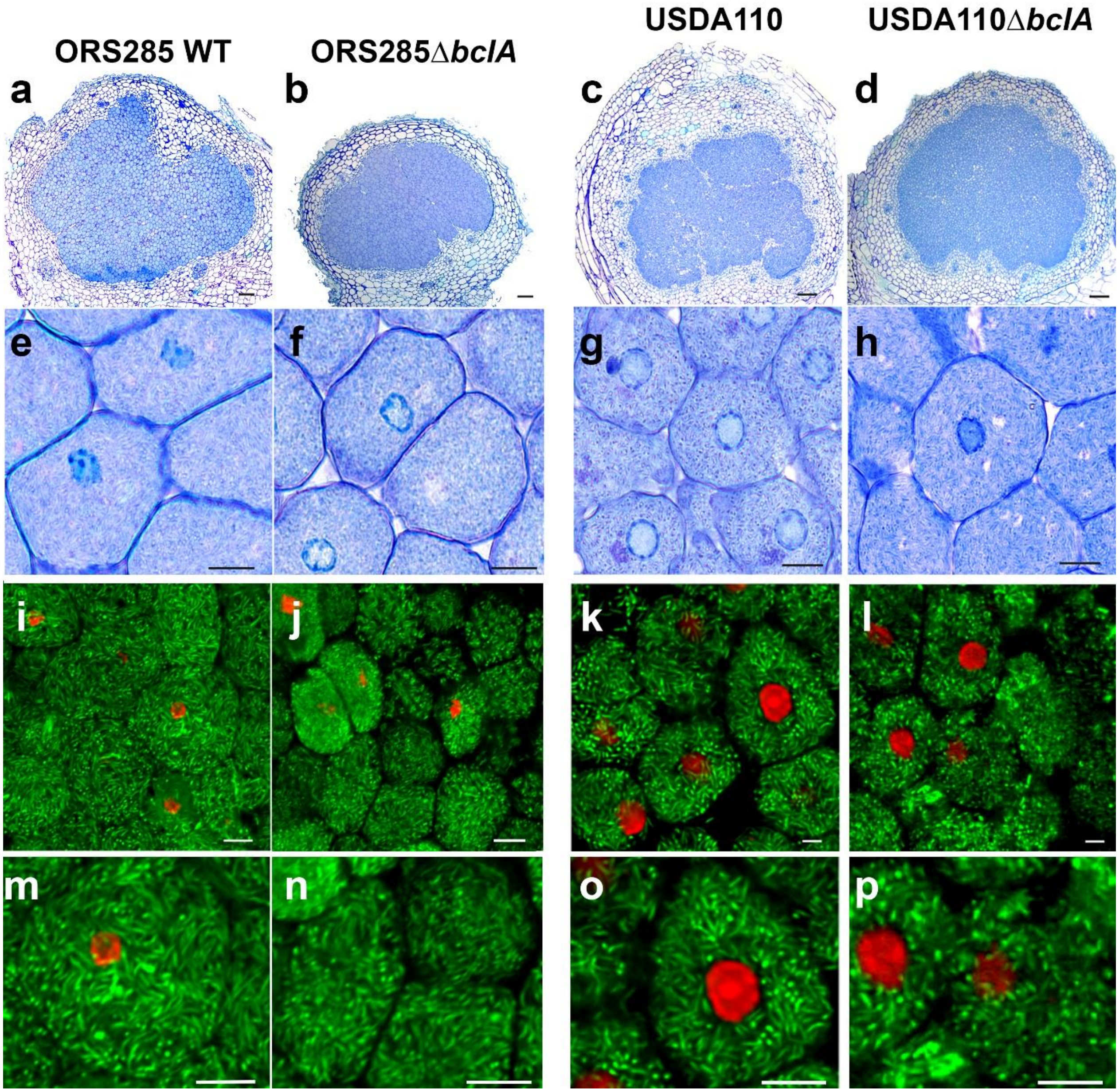

Fig. S1. Histology of *Aeschynomene afraspera* nodules infected with strains ORS285 or USDA110 and their *bclA* mutants. (**a-h**) Light microscopy of toluidine blue stained thin sections. (**i-p**) Confocal microscopy of fresh nodule sections stained with Live/Dead BacLight. Scale bars are 100 µm (a-d) or 10 µm (e-p).

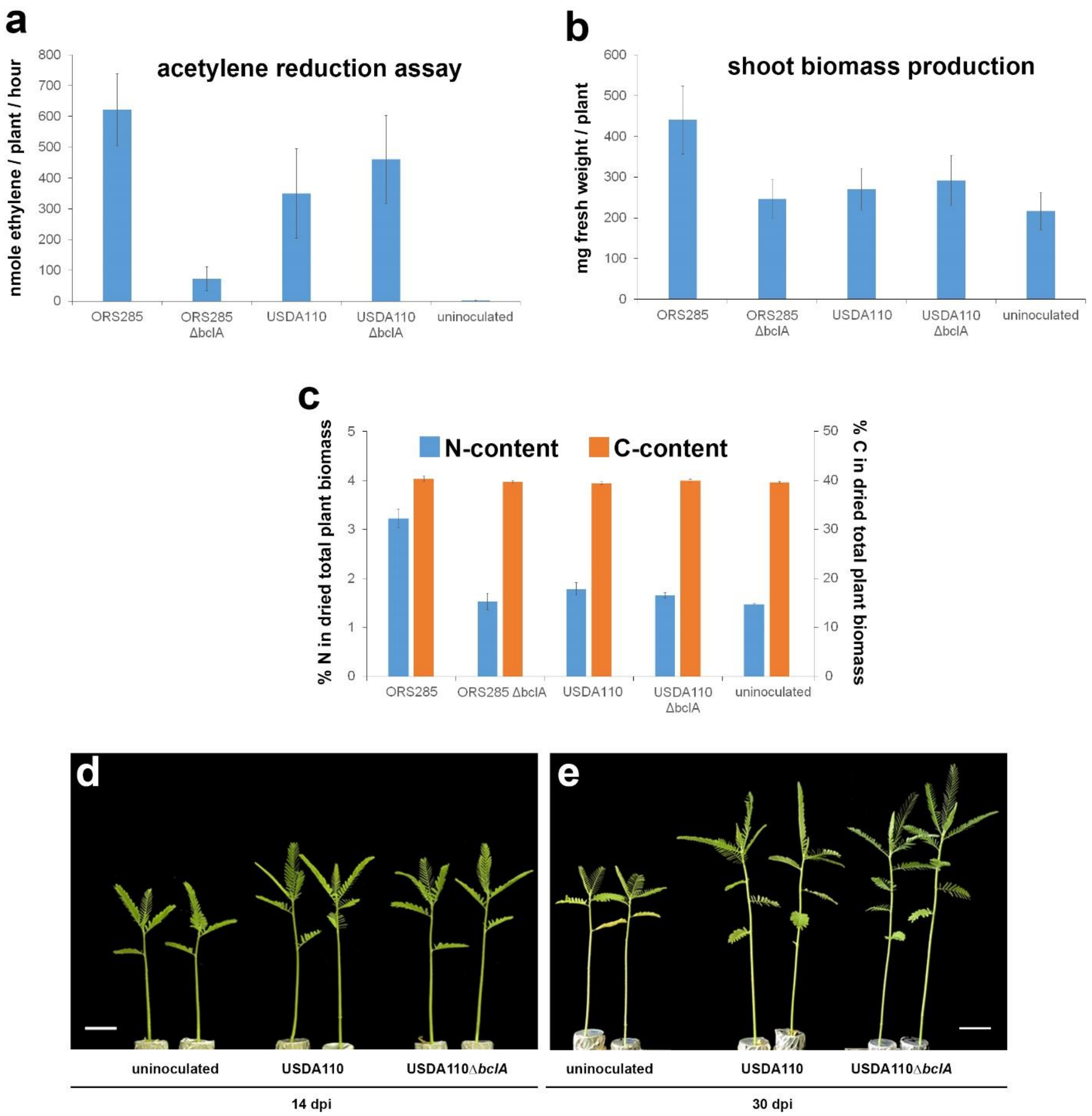

Fig. S2. Nitrogen fixation, plant growth and nitrogen content in *Aeschynomene afraspera* infected with strains ORS285, USDA110 and their *bclA* mutants. (**a**) Acetylene reduction assay of whole plants at 21 dpi. n=10 and error bars are standard deviations. (**b**) Shoot fresh weight of plants at 21 dpi. n=10 and error bars are standard deviations. (**c**) Nitrogen and carbon content of plants expressed as % of dry mass of de-nodulated plants. Each analysis was made on pools of three plants. n=2 or 3 and error bars are standard deviations. (**d**,**e**) Plant growth of uninoculated *A. afraspera* plants or plants inoculated with *B. diazoefficiens* USDA110 wild type or its *bclA* mutant at 14 dpi (d) or 30 dpi (e). Scale bars are 2 cm.

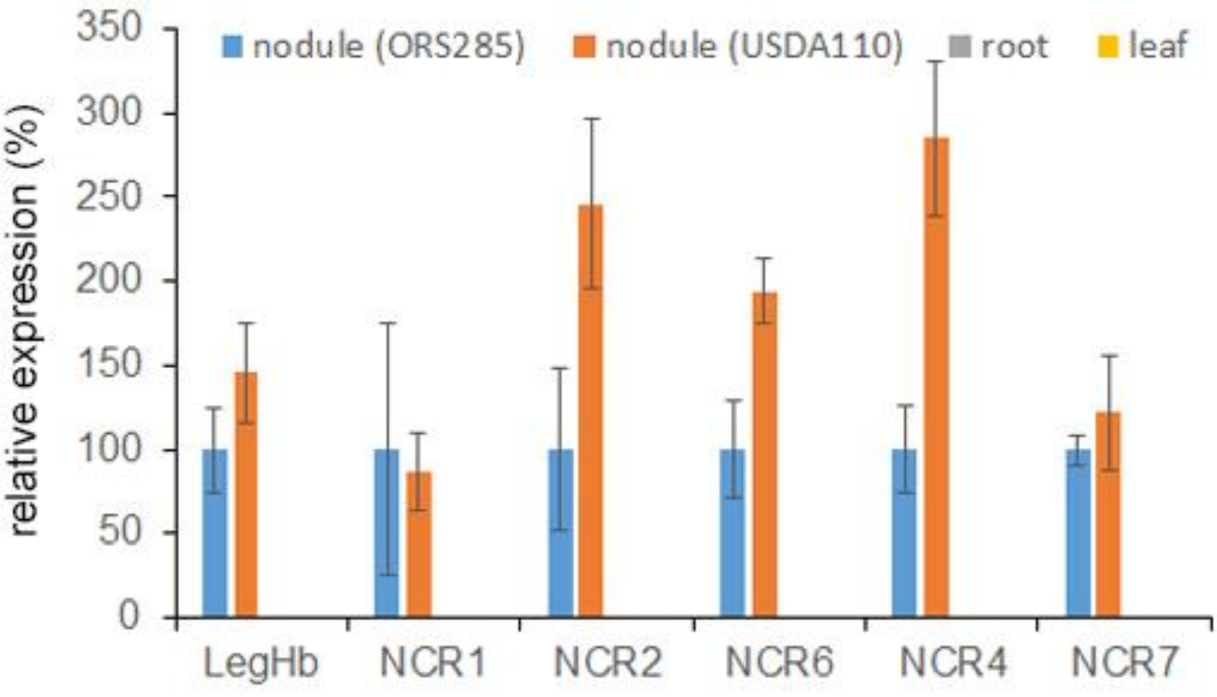

Fig. S3. Relative expression of 5 *NCR* genes and leghemoglobin in *A. afraspera* roots, leaves and nodules infected with *B. diazoefficiens* USDA110. The expression of each gene was normalized by the constitutive elongation factor 1α gene and set at 100% in the *A. afraspera* nodules infected with *Bradyrhizobium* strain ORS285. The expression of all six genes was below detection limit in the root and leave samples. Error bars are standard deviations of three biological repeats.
